# Neuro-psychiatric symptoms in directly and indirectly blast exposed civilian survivors of urban missile attacks

**DOI:** 10.1186/s12888-023-04943-1

**Published:** 2023-06-13

**Authors:** R. Saar-Ashkenazy, S. Naparstek, Y. Dizitzer, N. Zimhoni, A. Friedman, I. Shelef, H. Cohen, H. Shalev, L. Oxman, V. Novack, G. Ifergane

**Affiliations:** 1 Faculty of Social-Work, Ashkelon Academic College, 12 Ben Tzvi St, PO Box 9071, 78211 Ashkelon, Israel; 2 Department of Cognitive-Neuroscience, Ben-Gurion University of the Negev, Beer-Sheva, Israel; 3 Zlotowski Center for Neuroscience, Ben-Gurion University of the Negev, Beer-Sheva, Israel; 4 Department of Psychology Ben-Gurion, University of the Negev, Beer-Sheva, Israel; 5 Department of Psychology, Bar-Ilan University, Ramat Gan, Israel; 6 Clinical Research Center, Soroka University Medical Center, Beer-Sheva, Israel; 7 Department of Medical Neuroscience, Dalhousie University, Halifax, NS B3H4R2 Canada; 8 Department of Diagnostic Imaging, Soroka University Medical Center, Beer-Sheva, Israel; 9 Anxiety and Stress Research Unit, Faculty of Health Sciences, Ministry of Health, Ben-Gurion University of the Negev, Beer-Sheva, Israel; 10 Department of Psychiatry, Soroka University Medical Center, Beer-Sheva, Israel; 11 Department of Neurology, Soroka University Medical Center, Beer-Sheva, Israel

**Keywords:** Blast, TBI/mTBI, PCS, PTSD, DTI

## Abstract

**Background:**

Blast-explosion may cause traumatic brain injury (TBI), leading to post-concussion syndrome (PCS). In studies on military personnel, PCS symptoms are highly similar to those occurring in post-traumatic stress disorder (PTSD), questioning the overlap between these syndromes. In the current study we assessed PCS and PTSD in civilians following exposure to rocket attacks. We hypothesized that PCS symptomatology and brain connectivity will be associated with the objective physical exposure, while PTSD symptomatology will be associated with the subjective mental experience.

**Methods:**

Two hundred eighty nine residents of explosion sites have participated in the current study. Participants completed self-report of PCS and PTSD. The association between objective and subjective factors of blast and clinical outcomes was assessed using multivariate analysis. White-matter (WM) alterations and cognitive abilities were assessed in a sub-group of participants (*n* = 46) and non-exposed controls (*n* = 16). Non-parametric analysis was used to compare connectivity and cognition between the groups.

**Results:**

Blast-exposed individuals reported higher PTSD and PCS symptomatology. Among exposed individuals, those who were directly exposed to blast, reported higher levels of subjective feeling of danger and presented WM hypoconnectivity. Cognitive abilities did not differ between groups. Several risk factors for the development of PCS and PTSD were identified.

**Conclusions:**

Civilians exposed to blast present higher PCS/PTSD symptomatology as well as WM hypoconnectivity. Although symptoms are sub-clinical, they might lead to the future development of a full-blown syndrome and should be considered carefully. The similarities between PCS and PTSD suggest that despite the different etiology, namely, the physical trauma in PCS and the emotional trauma in PTSD, these are not distinct syndromes, but rather represent a combined biopsychological disorder with a wide spectrum of behavioral, emotional, cognitive and neurological symptoms.

## Introduction

Blast-explosion may cause a traumatic brain injury (TBI) with injury magnitude depending on factors such as blast energy and distance from the epicenter [24]. In mild-TBI (mTBI), a variety of physical, emotional, and cognitive symptoms are reported [76], and when these persist over three months, they are referred to as post-concussion syndrome (PCS, [63]. The pathophysiology underlying PCS is not-yet fully understood, since similar symptomatology is reported in other trauma victims [5, 11, 21, 49],see also reviews by, [19, 31, 59]) specifically in individuals with post-traumatic stress disorder (PTSD). PTSD can develop following exposure to a life-threatening event, if the individual felt in-danger, and suffers from re-experiencing, avoidance, hyper-arousal and negative alterations in cognition and mood [1, 2]. The peritraumatic response (i.e., feeling in danger, scared, or helpless at the time of the event) is a potential predictor for future PTSD [8, 9]. The high co-occurrence of mTBI and PTSD, especially in blast-related head injury [41, 46], questions the role played by the objective physical blast in causing such symptoms, and thus, should be further addressed.

Whereas other types of brain injury can be assessed using routine neuroimaging, PCS remains a challenge for such assessment, since the damage is associated with small, diffused, brain alterations (i.e., diffuse axonal injury, DAI) that cannot be reliably viewed on conventional computerized tomography (CT) or Magnetic Resonance Imaging (MRI) scans. DAI reflects white-matter (WM) damage from sustained forces acting on the brain, shearing axons and microscopic changes [30, 81] and can be visualized using diffusion-tensor imaging (DTI, [7, 26, 44, 53, 56, 83]. Microstructural changes in the brain were evident in rats following exposure [85] and repeated exposure [4] to blast wave, suggesting these might underlie PCS symptomatology in humans as well. Despite inconsistencies see reviews by [3, 52] numerous mTBI studies in humans report abnormal diffusion in frontal association, projection, and commissural fibers [29, 40, 44, 53, 56, 65, 66, 83] suggesting these might play a role in PCS. In addition, studies exploring populations exposed to single and repetitive traumatic events with [12, 45] or without [37, 38] mTBI have emphasized that these individuals show selective impairments in several cognitive and emotional domains [54],see also a review by [33]. In this manner, studies conducted in both concussive (mTBI) and non/subconcussive blast exposed participants, reported WM anatomical connectivity damage [74, 77]) and brain functional connectivity alterations [61, 62] which may explain the cognitive deficits exhibited by these participants.

The exposure to an explosion might lead to blast-related TBI [64], with damage to the brain tissue resulting of the blast-wave induced by the changes in the atmospheric pressure (primary blast injury), from objects in motion that might hit the head (secondary blast injury), or by the person being forcefully put in motion by the blast (tertiary blast injury) [75]. Importantly, these injuries are attenuated as a factor of the distance from the blast epicenter as well as the location of the explosion (i.e., open field or confined space) and whether the person was directly exposed to the blast or was protected by a shield [82].

Whereas blast-related TBI can occur in civilian settings, most studies in the field were conducted on military personnel leaving the etiology and prognosis of civilian blast-related TBI unaddressed [18]. Civilians exposed to constant stress (continued threats of blasts explosions as well as actual experiences such as missiles attacks) may show blast-related psychological response as described in military personnel [57]. This psychological response may include behavioral, emotional, cognitive and brain related dysfunctions that are not sufficiently defined by the standard measures of PTSD, highlighting the need to study the impact of blast in this population. Knowledge and improved understanding of blast induced long-lasting symptoms in civilians can help clinicians to facilitate treatment recommendations in this population.

The current study employed a multidisciplinary approach to examine PCS/PTSD unique and overlapping features in blast-exposed civilians. As Physical and mental exposures were examined by studying subjective self-reported symptomatology, objective measures of cognitive abilities, and objective WM alterations. We hypothesized that exposure to blast would lead to PCS/PTSD symptoms. Specifically, we hypothesized that blast impact would lead to objective WM alterations and cognitive deficits manifesting as PCS symptoms; and that mere exposure to blast would lead to a subjective emotional experience manifesting as PTSD symptoms.

## Methods

### Standard protocol approvals, registrations and patient consents

The study was approved by the Soroka University Medical Center Institutional Review Board (IRB) and all participants gave their written informed consent for participation. The current study is an ecological observational evaluation of clinical symptomatology, cognitive abilities, and neuroimaging in a non-clinical exposed population sample that was conducted in two phases.

## Phase 1

### Identification of recruitment sites

Following the completion of operation “Protective-Edge” in Israel (July–August 2014), rocket explosion sites were identified within the residential area of the city of Beer-Sheva using military official reports and media coverage, cross-linked, and verified with local citizens’ reports. Four explosion sites, sustaining only one BM-21 Grad missile explosion during the operation, and never sustaining similar events in the past 10 years, were chosen.

### Participants’ recruitment and screening

Participants’ enrollment began three weeks after the last rocket attack in the city of Beer-Sheva (August 2014) and lasted for ~ 2 years. Recruitment was performed from all four sites in parallel. Systematic door-to-door screening was performed by study personnel in and near the impact sites, starting from the vicinity of the explosion areas and up to a radius distance of 500 m from the explosion site. To maximize the number of respondents, door-to-door screening was performed not during working hours, namely, in the afternoon, or evening. Participants above the age of 18 who agreed to participate in the study were recruited. Individuals who were unable to sign an informed consent form and/or complete self-report questionnaires, as well as individuals who were not living in the same address or were not in their home during the missile attack, were excluded from the study. Upon meeting all inclusion criteria, participants were given an explanation about the study and signed an informed-consent form.

**Fig. 1:**
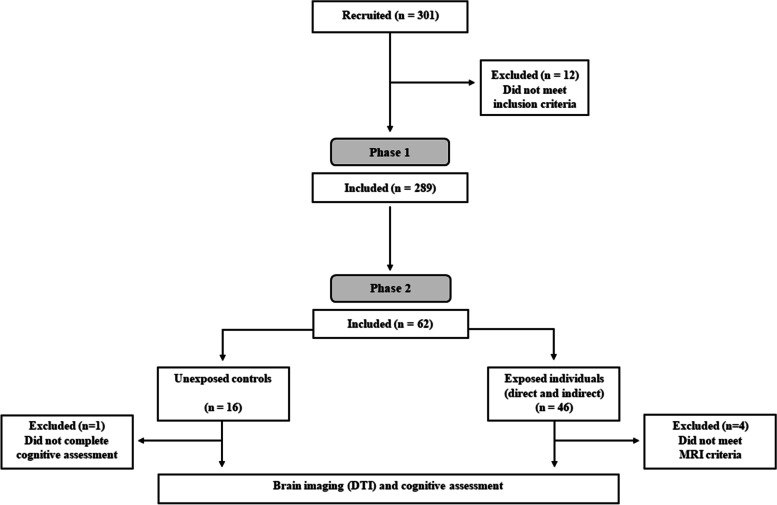
Consolidated standards of reporting trails (CONSORT) table describing chronologic study phases

Overall, 301 Beer-Sheva residents living in a range of 500 m from four impact sites were recruited to “phase 1” of the study, twelve of whom had met exclusion criteria, thus a total of 289 individuals participated in this phase of the study (see CONSORT table, Fig. 1). Of the 289 participants, 58 were directly blast-exposed participants and 231 indirectly blast-exposed participants (see below).

### Blast physical exposure assessment

Measures of residence-distance from the impact site were collected from maps, air photographs, and physical survey of the site. Following previous work on animal models and military personnel, the exposure of each participant to blast was evaluated according to his/her residency address, based on two parameters:

- (a) Distance from impact site– the aerial distance (in meters) of the residence building from the impact site.
- (b) Level of exposure – residency addresses were classified as “*directly-exposed*” when the residency building was in open “sightline” to the explosion site allowing full blast wave exposure, or “*indirectly-exposed*” when such a sightline was obstructed by other structures.

### Demographic and clinical symptom reports

Participants completed the following self-report questionnaires, with the presence of study personnel:

- Demographic (e.g., age, gender, employment history, as well as physical and mental health record) and rocket exposure questionnaires.
- The Rivermead Post-concussion syndrome Questionnaire (RPQ; [34]: a self-report measure of PCS assessing symptom severity following mTBI, consisting of 16 post-concussion symptoms (test–retest reliability ~ 0.89). Participants are asked to rate the severity of each item over the past 24 h, by comparing the severity of each symptom to how it was before the injury occurred. Responses are scored on a 5-point Likert scale ranging from '0' = “not experienced at all” to '4' = “severe problem”. As no universal benchmark for scoring the RPQ exists [79], we extracted three different scores to describe the severity of post concussive symptoms: a RPQ-Total scores: calculated by adding up the scores for all symptoms, except for ratings of 1 which meant that the symptoms had not changed since the injury. A total sum score equal or higher than 12 implies PCS,b RPQ-3: sum scores of the first three items in the questionnaire (Headache, Nausea/Vomiting, Dizziness, requiring one or more symptom to reach a cutoff,and c RPQ-13: sum scores of the latter 13 items (excluding the first three items. The last two scores relate to distinct PCS clusters,where the RPQ-3 describes more early, predominantly physical symptoms, the RPQ-13 describes more late, predominantly psychological symptoms [20]. The current study employed a widely-used Hebrew translated version of the RPQ (see also [35, 50].)
- The Post-Traumatic Diagnostic Scale for DSM IV [22, 23] is a self-report measure of PTSD related to a single identified traumatic event (Cronbach’s Alpha reliability = 0.92). The PDS assesses the presence and nature of the trauma (item A1), the emotional subjective response during the event (item A2), as well as symptom severity (i.e., items B-D). The subjective response (A2) includes a positive response to at least one of the following questions: “Did you feel in danger?”, “Did you feel helpless?”, and “Did you feel scared?”. Symptom severity, on each of the 17 DSM-IV PTSD symptoms, is rated on a scale from 0 (“not at all/ only one time”) to 3 (“5 or more times a week/almost always”), with total scores ranging from 0–51. In the current study we examined both the subjective response and overall severity. The current study employed a widely-used Hebrew translated version of the PDS see also [14, 25, 28].

### Statistical analysis

Data were analyzed using IBM Statistical Package for Social Sciences (SPSS, version 24^©^).

Normality was assessed for all variables prior to conducting statistical comparisons. Variables with non-normal distribution are presented as medians and inter-quartile range (IQR) and were compared via Man-Whitney U test. Descriptive statistics of demographic and clinical characteristics for nominal variables are presented as n (%) and were compared via Pearson’s Chi-square.

Multivariate Generalized Linear Mixed Models (GLMM) were then performed to assess the degree of association between physical-objective (e.g., direct/indirect exposure, distance from blast) and emotional-subjective blast-related factors (e.g., DSM-IV stressor criteria A2) to clinical outcomes – PCS and PTSD symptoms’ severity, assessed by RPQ and PDS respectively. Models were adjusted to demographic characteristics, medical history of head trauma, and time passed the explosion.

## Results

The median aerial distance from the blast-site in the directly exposed group was 35.5 m (IQR = 10–85.5). In the indirectly exposed group, the median aerial distance from the blast-site was 218 (IQR = 175–293). The two exposure groups significantly differed in the median aerial distance from the blast impact site (z = -10.26, *p* = 0.000).

Tables 1a and 1b depict demographical and clinical characteristics of the study population, as well as experience related factors of the blast exposure and the primary outcomes.

**Table 1:** Population characteristics by level of blast exposure (direct vs. indirect)

**Population characteristics**	Total *N* =289 (100%)	Direct exposure *N* =58 (20%)	Indirect exposure *N* =231 (80%)	***p*-value**
**A. Demographical characteristics**				
Age at time of explosion ^a^	40.41 [31.35–55.41]	46.55 [30.04–66.44]	39.81 [31.66–53.52]	0.151
Gender (Males) ^b^	104 [36%]	21 [37%]	83 [36%]	0.900
**Marital status (married) **^**b**^	**209 [72%]**	**31 [55%]**	**178 [80%]**	** < 0.001**
**Education years **^**a**^	**15 [12–17]**	**12 [12–16]**	**15 [12–17]**	**0.041**
**Employed **^**b**^	**190 [66%]**	**30 [55%]**	**160 [73%]**	**0.010**
**Past military service **^**b**^	**183 [63%]**	**28 [50%]**	**155 [74%]**	**0.001**
History of head trauma ^b^	54 [19%]	10 [18%]	44 [20%]	0.740
**B. Clinical characteristics**				
**Emotional experience during explosion (A2 criteria)**				
**Felt danger during blast **^**b**^	**83 [29%]**	**24 [41%]**	**59 [26%]**	**0.017**
Felt helplessness during blast ^b^	76 [26%]	18 [31%]	58 [25%]	0.359
Felt scared during blast ^b^	92 [32%]	23 [40%]	69 [30%]	0.153
**Post-exposure symptoms’ severity**				
PTSD (PDS score) ^a^	1 [0–7]	3 [0–8]	1 [0–6]	0.076
Post-Concussion (RPQ score) ^a^	2 [0–18]	1 [0–17]	2 [0–18]	0.323
RPQ3 score ^a^	0 [0–2]	0 [0–2.25]	0 [0–2]	0.776
RPQ13 score ^a^	2 [0–15]	1 [0–14]	2 [0–16]	0.306

*Note.* Table 1 shows population characteristics including demographic characteristics (A), parameters assessing the emotional experience of the blast and post exposure symptom severity depicted by PDS and RPQ scores (B)

^a^ Variables are depicted by Median and [IQR] and compared via Man-Whitney U test

^b^ Variables are depicted by n (%) and compared via Pearson’s Chi-square;. Statistically significant results appear in bold

### Participants

Statistical analysis was performed on 289 subjects who met all inclusion criteria. Univariate analysis comparing the direct- versus indirect-exposure groups yielded no statistically significant differences in age, gender, past traumatic events, or history of head trauma between the groups (*p* > 0.05). In addition, in both groups, no injuries were reported following the blast explosions and no significant difference was evident between the two exposure groups in reporting whether they felt the shock-wave (χ^2^(1) = 0.183, *p* = 0.669). Significant differences between groups were evident in marital status, years of education, employment and past military service (see Table 1a).

### Clinical symptomatology

Cronbach’s Alpha reliability analysis resulted in acceptable values for both self-report questionnaires for PTSD, as measured via the PDS questionnaire, and PCS symptom severity, as measured via the RPQ questionnaire (0.931 and 0.931, respectively). A significant linear correlation was observed between PTSD symptom severity and PCS symptom severity, *r* = 0.704, *N* = 289, *p* = 0.000. This correlation remained significant when computed separately for exposure groups; r_indirectly-exposed_ = 0.693, *N* = 231, *p* = 0.000 and r_direct-exposed_ = 0.778, *N* = 58, *p* = 0.000, with no significant difference in the strength of the correlation between exposure groups (z = -1.242, *p* = 0.107). As can be seen in Table 1b, PTSD symptom severity was within the lower end of the mild range. PCS symptom severity was also within the low range for early and late symptom clusters. No statistically significant differences were found between directly- and indirectly-exposed participants in subjective reports of PTSD or PCS symptom severity. Notwithstanding, the DSM-IV stressor criteria A2, corresponding to the peritraumatic experience, significantly differed between exposure groups, with directly-exposed individuals reporting higher levels of feeling-in-danger during the explosion (41% vs. 26%, *p* = 0.017).

Multivariate analysis via GLMM using physical-objective (e.g., direct/indirect exposure, distance from blast) and emotional-subjective blast-related factors, adjusted for demographic and clinical parameters, showed several risk factors for PCS and PTSD (see Table 2). Female gender (F(1, 245) = 9.892, *p* = 0.000; F(1, 245) = 14.980, *p* = 0.000), history of head trauma (F(1, 245) = 7.169, *p* = 0.008; F(1,245) = 3.984, *p* = 0.047) and time since the explosion (F(1, 245) = 8.765, *p* = 0.003; F(1, 245) = 6.732, *p* = 0.01) were risk factors for the development of both PCS and PTSD symptoms, respectively. Un-married individuals (F(1, 245) = 4.353, *p* = 0.038) and individuals who reported feeling scared at the time of the blast-explosion (F(1, 245) = 4.133, *p *= 0.043) were at higher risk to develop PCS.

**Table 2:** GLMM coefficients for PCS and PTSD severity

**Factor**	**Coefficient**	**t**	***p*-value**	**CI (95%)**	
				**Lower**	**Upper**
**A. GLMM coefficients for PCS severity**					
**Gender (Male)**	**-0.757**	**-3.145**	**.002**	**-1.231**	**-0.283**
Age	0.009	1.190	.235	-0.006	0.024
Employment (Employed)	-0.173	-0.664	.507	-0.685	0.340
Past military service (Served)	-0.429	-1.755	.081	-0.911	0.052
**Marital status (Married)**	**-0.498**	**-2.033**	**.043**	**-0.981**	**-0.015**
**History of head trauma (Yes)**	**0.742**	**2.678**	**.008**	**0.196**	**1.288**
**Time passed the explosion (weeks)**	**0.012**	**2.961**	**.003**	**0.004**	**0.020**
Exposure (Direct exposure)	-0.473	-1.447	.149	-1.118	0.171
Distance (Meters)	0.000	-0.292	.770	-0.003	0.002
Safe-zone (Yes)	0.049	0.180	.857	-0.482	0.579
A2_Criteria: Felt danger	-0.071	-0.184	.854	-0.829	0.687
A2_Criteria: Felt helpless	-0.379	-1.003	.317	-1.123	0.365
**A2_Criteria: Felt scared**	**0.878**	**2.086**	**.038**	**0.049**	**1.707**
**B. GLMM coefficients for PTSD severity**					
**Gender (Male)**	**-0.931**	**-3.870**	**.000**	**-1.405**	**-0.457**
Age	0.005	0.661	.509	-0.009	0.019
Employment (Employed)	-0.332	-1.309	.192	-0.831	0.167
Past military service (Served)	-0.401	-1.715	.088	-0.862	0.060
Marital status (Married)	-0.432	-1.830	.068	-0.897	0.033
**History of head trauma (Yes)**	**0.526**	**1.996**	**.047**	**0.007**	**1.045**
**Time passed the explosion (weeks)**	**0.010**	**2.595**	**.010**	**0.002**	**0.018**
Exposure (Direct exposure)	-0.293	-0.927	.355	-0.917	0.330
Distance (Meters)	-0.002	-1.483	.139	-0.004	0.001
Safe-zone (Yes)	-0.256	-1.012	.313	-0.753	0.242
A2_Criteria: Felt danger	0.418	0.934	.351	-0.463	1.299
A2_Criteria: Felt helpless	-0.112	-0.256	.798	-0.974	0.750
A2_Criteria: Felt scared	0.224	0.533	.594	-0.603	1.051

*Note. GLMM* Generalized linear mixed model, *PCS* Post concussion syndrome, *PTSD* Posttraumatic stress disorder, *CI* Confidence interval; Statistically significant results appear in bold

To summarize, the peritraumatic experience differed between the exposure groups. Furthermore, we identified several risk factors for both PCS and PTSD symptomatology; these include female gender, history of head trauma and time passed from the explosion (for both syndromes); as well as marital status and feeling scared (for PCS).

## Phase 2

### Methods

#### Participants’ recruitment and screening

Participants (both exposed and unexposed, see below) were parallelly recruited to the second phase of the study which took place approximately ~ 1 year after the operation. Of the 289 participants who took part in “Phase 1”, participants were telephonically approached and were offered to participate in the second study phase which included cognitive and neuroimaging testing. Those who were willing to participate and presented no contraindications to MRI, were invited to take part in the next phase of the study (“Phase 2”) at Soroka University Medical Center, Beer-Sheva, Israel. Overall, 46 phase 2 individuals completed a cognitive assessment battery; of those, 42 eligible participants underwent MRI (see CONSORT table, Fig. 1). In addition, a control group of 16 non-blast-exposed age, gender and education matched individuals was recruited (all participated in the imaging phase, 15 completed cognitive tests). These participants were not exposed to any of the four blast explosions described in phase 1. Following definition of participants in phase 1 (i.e., directly/indirectly-exposed), control group participants were defined “*unexposed*”.

#### Cognition

Cognitive functioning was assessed using a computerized test battery (NeuroTrax Corp., Newark, NJ) in Hebrew. This battery is a patented, scientifically validated computerized cognitive assessment system that assesses performance across an array of cognitive domains. Participants’ performance is compared to age-and-education matched controls and then normalized, yielding scores with an average of 100 and standard deviation (SD) of 15. Scores within 1 SD of the average (the 85–115 range) represent normal or average abilities. The system has been validated with several populations such as mild cognitive impairment patients – MCI [16, 17], Attention Deficit Hyperactivity Disorder (ADHD, [39, 69] schizophrenia [60] and depression [58] and is recognized as a standard research tool for cognitive assessment.

#### MRI and DTI data acquisition & processing

Scans were acquired using a 3-Tesla scanner (Ingenia, Philips Medical Systems), and included: Anatomical scans (3D-TFE T1-w, TR/TE = 8.1/3.7 ms, field of view (FOV) 256 mm, 150 slices); T2-w and FLAIR sequences were acquired as part of a clinical routine. DTI data were acquired on the same scanner using a single-shot echo-planar imaging (EPI) sequence with SENSE parallel imaging (reduction factor = 2.5). 60 Axial slices of 2.0 mm (zero-gap) thickness were acquired parallel to the anterior–posterior commissure line (AC-PC). The in-plane acquisition resolution was 2.88 × 3.58 mm. The DTI data were acquired along 33 directions with b = 1000 s/mm^2^. A TR/TE = 9000/106 ms (ms) was used without cardiac gating for a total acquisition time of 6:21 min for each dataset. All fiber tracking was performed using mrDiffusion, an open source package written by the Vision, Imaging Science and Technology Activities (VISTA) lab at Stanford, CA, USA (http://web.stanford.edu/group/vista/cgi-bin/wiki/index.php/Software), and in-house Matlab (Mathworks, Natick, MA) scripts. In total, 4 average diffusion parameters (fractional anisotropy (FA), mean diffusivity (MD), radial diffusivity (RD) and axial diffusivity (AD)) were calculated for each tract (*n* = 20, automatically defined by the mrDiffusion package) and for each participant.

#### Statistical analysis

Normality was assessed for all variables prior to conducting statistical comparisons. Variables with non-normal distribution are presented as medians and inter-quartile range (IQR) and were compared via Man-Whitney U test. Descriptive statistics of demographic and clinical characteristics for nominal variables are presented as n (%) and were compared via Pearson’s Chi-square.

Due to contradicting findings in literature regarding the nature and specificity of WM alterations in mTBI/PCS, we carried out whole brain diffusion analysis for 20 fibers and we then compared diffusion parameters between directly, indirectly and non-exposed participants using Kruskal–Wallis tests.

## Results

Table 3 depicts demographical, clinical characteristics and cognitive outcomes of participants that underwent “phase 2” and performed neuroimaging and cognitive evaluation for the exposed and unexposed (control) groups.

**Table 3:** Population characteristics by exposure to blast and level of exposure (direct vs. indirect) for cognition and imaging sub population

Population characteristics	Direct exposure *N* = 13 (22%)	Indirect exposure *N* = 29 (50%)	No exposure *N* = 16 (28%)	Exposed vs. non-exposed (*p*-value)	Direct vs. indirect (*p*-value)
**Age at interview **^**a**^	**30.6 [26.2–49.6]**	**49.1 [32.5–67]**	**59.5 [41.2–62.5]**	**0.124**	**0.02**
Gender (Males) ^b^	5 [38.5%]	10 [34.5%]	9 [56%]	0.156	0.804
Education ^b^					
Elementary	1 (8%]	1 [4%]	0		
High school	5 [38.5%]	12 [43%]	6 [40%]	0.665	0.839
Academic	7 [53.5%]	15 [54%]	9 [60%]		
Past military service ^b^	10 [77%]	20 [69%]	11 [85%]	0.340	0.598
Chronic medication ^b^	3 [23%]	13 [48%]	3 [23%]	0.269	0.130
History of head trauma ^b^	4 [31%]	6 [21%]	4 [29%]	0.722	0.478
**Post-exposure symptoms’ severity**					
**PTSD (PDS score) **^**a**^	**3 [0.5–13.5]**	**3 [0–15.5]**	**0 [0–4]**	**0.033**	**0.966**
**RPQ3 **^**a**^	**0 [0–3.5]**	**0 [0–0]**	**0 [0–0]**	**0.047**	**0.257**
RPQ13 ^a^	**4 [0–19]**	**0 [0–2]**	**0 [0–0]**	**0.005**	**0.176**
Post-Concussion (RPQ score ^a^	**5 [0–24]**	**0 [0–2]**	**0 [0–0]**	**0.005**	**0.176**
**Cognition**^c^					
Memory ^a^	103 [97–106]	99 [84–105]	102 [90–105]	0.760	0.211
Executive function ^a^	107 [96–112]	104 [97–111]	98 [85–105]	0.168	0.693
Visual spatial ^a^	105 [89–116]	97 [86–111]	104 [90–114]	0.448	0.219
Verbal function ^a^	107 [99–113]	101 [69–110]	103 [96–109]	0.831	0.249
Attention ^a^	104 [96–107]	102 [92–109]	99 [93–106]	0.737	1.000
Information ^a^ processing speed	105 [96–118]	105 [95–109]	97 [91–109]	0.283	0.560
Motor skills ^a^	101 [97–107]	104 [100–108]	102 [96–111]	0.976	0.486
Global cognitive ^a^ assessment	104 [100–108]	101 [90–106]	98 [92–105]	0.556	0.154

*Note.* Table 3 shows demographic and clinical characteristics for sub-population divided by direct exposure, indirect exposure and no exposure to blast. Univariate comparison was held between exposed vs. non-exposed participants and direct vs. indirect levels of explosion

^a^ Variables are depicted by Median and [IQR] and compared via Man-Whitney U test

^b^ Variables are depicted by n (%) and compared via Pearson’s Chi-square. Statistically significant results appear in bold

^c^ Data for the no-exposure group is reported for 15 participants

### Participants

Statistical analysis was performed on 58 subjects who met all inclusion criteria. Comparing direct (*n* = 13), indirect-exposed (*n* = 29) and non-exposed participants (*n* = 16) yielded a significant difference in age between groups between directly exposed and indirectly exposed participants (z = -2.326, *p* = 0.02). No other significant differences in demographic characteristics were found (see Table 3).

### Clinical symptomatology

Post-exposure symptom severity analysis showed significant differences between exposed and unexposed participants in both PTSD and PCS symptoms (*p* = 0.03 for PDS and *p* = 0.047 for RPQ-3, and *p* = 0.005 for RPQ13 and RPQ total, respectively) with exposed participants showing higher levels of symptom severity compared to unexposed participants. No other significant differences in clinical characteristics were found (see Table 3).

### Cognition

Directly exposed (*n* = 13), indirectly exposed (*n* = 29) and unexposed (controls, *n* = 15) participants completed the NeuroTrax battery. There were no significant differences in cognitive abilities between the groups (Table 3).

### Imaging

**Fig. 2:**
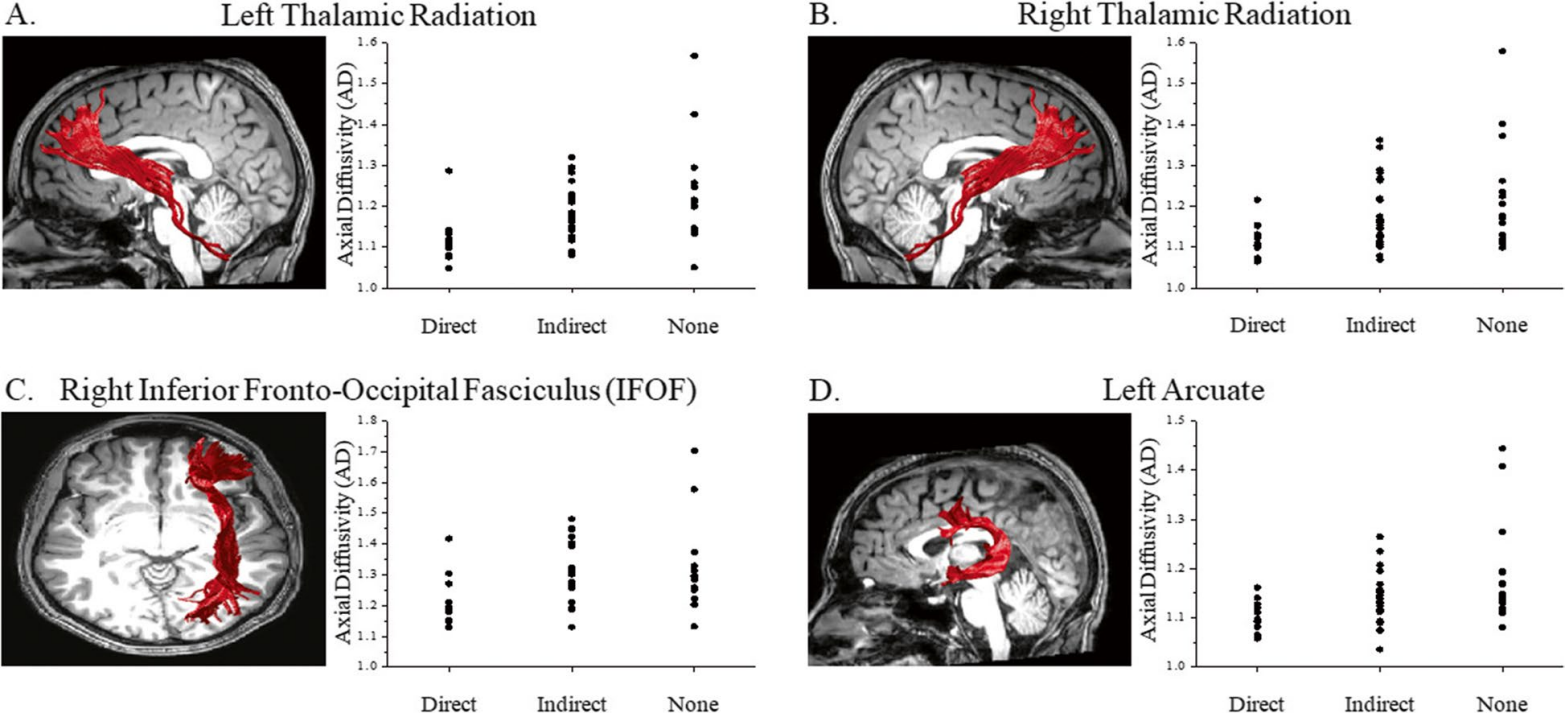
Diffusion imaging results for selected fibers. Significant differences were found in the left (**A**) and right (**B**) thalamic radiation, Inferior Fronto-Occipital Fasciculus (IFOF, **C**) and left arcuate (**D**)

In order to determine WM brain differences between groups, Kruskal–Wallis tests were performed. This analysis resulted in significant differences between the three exposure groups (direct, indirect and non-exposed participants) in the following tracts and parameters: the left cortico-spinal fasciculus FA values (H = 10.822, *p* = 0.004), with directly exposed participants significantly differing from non-exposed participants (*p* = 0.003); the left and right thalamic radiation MD values (left: H = 6.55, *p* = 0.037), right: H = 7.079, *p* = 0.029) with directly exposed participants significantly differing from non-exposed participants (*p* = 0.031, *p* = 0.024, respectively); the left and right cingulum cingulate MD values (left: H = 6.529, *p* = 0.038, right: H = 6.086, *p* = 0.047), with indirectly exposed participants significantly differing from non-exposed participants (*p* = 0.049, *p* = 0.041, respectively). The most predominant differences were evident in the values of the AD parameter; these include differences in the left thalamic radiation (H = 13.605, *p* = 0.001), with directly exposed participants, significantly differing from both non-exposed participants (*p* = 0.000) and indirectly exposed participants (*p* = 0.015) with a similar pattern of differences observed also for the right thalamic radiation (H = 8.485, *p* = 0.014) with directly exposed participants significantly differing from both non-exposed participants (*p* = 0.018) and indirectly exposed participants (*p* = 0.036). Additional differences were also observed in the left cortico-spinal fasciculus (H = 10.035, *p* = 0.006) with non-exposed participants differing from both directly exposed (*p* = 0.007) and indirectly exposed (*p* = 0.044) participants. Both the left cingulum cingulate (H = 6.541, *p* = 0.038) and the right cingulum cingulate (H = 7.141, *p* = 0.028) showed a similar pattern of differences, with directly exposed participants significantly differing from non-exposed participants (*p* = 0.044, *p* = 0.046, respectively). Also, the AD values of the right inferior fronto-occipital fasciculus (IFOF) differed between groups (H = 7.157, *p* = 0.027) with directly-exposed participants differing from indirectly exposed participants (*p* = 0.04). In the superior longitudinal fasciculus (SLF), differences were evident in the right side H = 7.893, *p* = 0.019) and showed that directly exposed participants significantly differed from both non-exposed participants (*p* = 0.043) and indirectly exposed participants (*p* = 0.027). Lastly, differences were evident in the left arcuate (H = 8.952, *p* = 0.011) with directly exposed participants, significantly differing from non-exposed participants (*p* = 0.008). Diffusion imaging results for selected fibers appears in Fig. 2.

To summarize, exposed participants (direct- and indirect) showed higher subjective symptomatology compared to unexposed controls. Furthermore, when comparing direct- and indirectly-exposed individuals, directly-exposed individuals presented hypoconnectivity in specific WM tracts.

## General discussion

In the current study we used an ecological approach to explore the association of two major components of an explosion in an urban setting: an objective component- the physical impact of the blast; and a subjective component- the emotional experience. We combined self-reports of PCS and PTSD symptomatology with objective measurements of cognitive functioning and alterations in WM neuro-anatomical connectivity. The study population is a community based, non-clinical cohort, recruited based on place of residence and vicinity to the impact site. This setting allows testing both direct and indirect blast-induced psychological and neurological pathologies. These alterations have been highlighted by studies exploring mental health effects following both direct and indirect exposure to traumatic events, showing a sharp increase of PTSD-like symptoms that rapidly decline following several months to a lower and stable rate [55].

The results of phase 1 showed that PCS and PTSD self-reported symptom severity did not differ between directly- and indirectly-exposed civilians. However, the subjective component of the exposure (the peritraumatic response, DSM-IV criteria A2) was significantly elevated in those directly-exposed. Regardless of levels of exposure, we identified several risk factors for the development of PCS and PTSD symptoms. These included female gender, history of head trauma and time passed from the explosion (for both syndromes); as well as marital status and feeling scared (for PCS).

In phase 2 we found that blast exposure was significantly associated with both PTSD and PCS symptomatology, as both direct and indirect-exposed civilians (compared to unexposed controls) reported higher symptom severity. Finally, directly-exposed civilians showed hypoconnectivity in specific WM tracts. Taken together, our findings suggest that civilians, who were exposed to blast, reported higher PTSD and PCS symptomatology compared to unexposed civilians. Among exposed civilians, those who were directly-exposed reported significantly higher feeling of danger, and presented specific WM hypoconnectivity.

Our findings highlight differences between exposed and unexposed civilians in the subjective experience to the traumatic event. In studies on clinical populations, subjective experience of fear during the event was associated with later development of PTSD symptoms [8]. Other studies show that the peritraumatic response that occurs at the time, or immediately after the trauma, is a predictor of later PTSD [9]. Thus, although directly exposed civilians did not report higher PTSD symptomatology overall, one of the major predictors for later PTSD was elevated in this group.

Following our hypothesis, WM connectivity was modulated by exposure to the impact site, with specific WM diffusion alterations occurring in direct-exposed civilians solely. In the mTBI literature, multifocal WM lesions causing disruptions of the axolemma and neurofilament organization are considered as representations of microscopic changes that result from sustained acceleration and deceleration forces associated with blast exposure [30, 81]. Lower AD values are thus considered indicative of axonal injury [10, 71, 72] and may imply on differential blast impact in residences from different exposure levels. Unlike our hypothesis, these WM diffusion alterations did not correlate with clinical symptoms or with cognitive abilities. Several meta-analyses and reviews also demonstrated inconsistencies regarding the specificity of DTI findings and their correlation with clinical/behavioral symptoms [15, 80]. Studies on blast exposure, primarily in military populations, also resulted in conflicting findings [13, 36, 42, 47]. Our imaging results demonstrate that WM DTI alterations occur following the exposure to blast explosion in an urban environment. Importantly, the brain alterations described in the current study are in-line with studies reporting blast-induced TBI alters the fear/anxiety neurocircuitry on the neuromolecular level and that these alterations resemble the changes that are often reported in PTSD patients (reviewed in [78].

The unique study setup and design allowed us to explore the association between PCS symptomatology and the level of exposure to the physical effect of the explosion, e.g., the blast wave. The symptoms of PCS are usually considered to be sequelae of a head injury, and if this is the case, we would have expected them to be most prevalent and severe close to the epicenter of the blast wave. Namely, that directly-exposed civilians would show higher PCS symptoms. Our data demonstrated no such association, questioning the role played by the physical blast in causing the symptoms. Previous work has questioned the attribution of PCS symptomatology to physical head trauma. While prior research examining the effects of blast-exposure on neuropsychological function have attributed the presence of neuropsychological sequelae to TBI/mTBI, other studies have showed a complex pattern [12] with post-concussive symptoms were found to occur at similar rates in trauma victims who sustained mTBI and in those who did not [48]. Soldiers with mTBI were significantly more likely to report a high number of somatic and post-concussive symptoms than soldiers with other injuries. However, the impact of these post-concussive symptoms became non-significant after PTSD and depression were considered [27]. mTBI is associated with high rates of PTSD in both military and civilian populations, and especially in blast related head injury [41]. Recent studies examining blast-exposed neuropsychological symptoms observed in veterans show that these impairments may be better explained by depression and PTSD-like psychiatric symptoms than by blast exposure, [43, 46, 54, 73]. Taken together, it is not clear if the physical impact or the psychological trauma is associated with the syndrome. In our cohort, female gender, history of head trauma, time passed since the exposure, marital status and the subjective feeling of feeling scared at the time of the explosion, all served as risk factors for PCS symptomatology. Importantly, these risk factors were identified in previous studies conducted on military personnel that were exposed to blast explosions [68, 84].

The coexistence of a highly similar set of objective and subjective risk factors for both disorders, and the difficulty to distinguish between the effects of physical and psychological trauma is not new and has roots dating back to the historical entity known as “shell shock” (see [19, 21, 31]. This discussion highlights the complexity in differential diagnosis between the two disorders and may suggest that they are not distinct symptom clusters. Alternatively, the current findings suggest they represent a spectrum of a combined biopsychological disorder that may develop as a result of a physical injury, or as a result of multiple objective and subjective trauma related factors. This view is supported by animal studies showing the development of PTSD-related behaviors in rats exposed to blast, in the absence of a psychological stressor (as exposure to blast is performed under general anesthesia) suggesting the existence of blast-induced PTSD syndrome [59]. This view is additionally supported by human studies asserting that psychiatric symptoms (e.g. depression and PTSD) rather than objective component of blast exposure, better explains the neuropsychological symptoms reported by blast-exposed individuals [12, 27, 41, 43, 46, 48, 54, 73].

Advances in neuroscience, and in unraveling the brain mechanisms underlying PCS and PTSD, have made the historical discussion [67] about “neurogenic” versus “psychogenic” etiology obsolete. Representing different etiologies and models of brain activity, PCS and PTSD are neither psychogenic nor neurogenic [51]. Rather, we suggest they should be referred to as a combined biopsychological disorder with a variety of behavioral, emotional, cognitive and neurological symptoms. Our study attempts to approach this issue from a multidisciplinary approach, dissecting head trauma into two elements, physical (the blast) and psychological (the experience), and evaluating the contribution of both to symptomatology. This kind of dissection is difficult in most cohorts and study designs, since neither the impact nor the experience can be quantified, but when it is feasible, it may allow better understanding of the disorders.

The current study has several limitations. The study evaluates blast-related symptomatology in a unique and innovative design. Nevertheless, it is a retrospective study, and therefore its generalizability is limited. It is also limited by the fact that recruitment was performed during an extended period of time, and that imaging was performed on a single time point in a sub-group of the study cohort. Although it provides further insight into the relationship between PCS and PTSD, due to its cohort retrospective nature it cannot establish causality between PCS and PTSD to blast exposure. Future studies enabling repeated measures of subjective and objective aspects of blast exposure, might provide further insight into the trajectory of these two syndromes over time. Such studies would also allow the establishment of biomarkers that would help guide diagnosis and prevention.

Taken together, the current findings suggest that exposure to blast affects both objective, neuro-physical (i.e., WM alterations) and subjective, mental (i.e., feeling in danger/scared) aspects. Differences in emotional experience were limited to the peritraumatic experience and not to symptom severity overall. It is possible that these specific, but significant, differences are sub-clinical manifestations of the full-blown PCS/PTSD disorder. Furthermore, it is possible that the observed alterations in WM neuro-anatomical connectivity and mental experience will predict future escalation, as the strength of the peritraumatic response is reported as a predictor of future development of PTSD [8, 9], and WM alterations in similar tracts was reported to be associated with PTSD symptom severity and cognitive deficits [32, 70]. Overall, our findings suggest that even if they are not clinically diagnosed, civilians who are directly exposed to explosion blast, suffer from mental and WM neuro-anatomical changes and should thus be considered a population at risk for the future development of PTSD and/or PCS. To the best of our knowledge, this is the first systematic report of the long-term effects of blast-related PCS/PTSD in civilian population. While some of the findings were similar to those reported in military populations, others were somewhat different thus highlight the importance of low-level blast exposure (see review by [6] and blast consequences in civilians [64] and the need for interdisciplinary collaborations in order to study them carefully.

## Data Availability

The datasets used and/or analysed during the current study are available from the corresponding author on reasonable request.

## Abbreviations

TBI: Traumatic brain injury
mTBI: Mild traumatic brain injury
PCS: Post-concussion syndrome
PTSD: Post-traumatic stress disorder
WM: White-matter
DAI: Diffuse axonal injury
CT: Computerized tomography
MRI: Magnetic resonance imaging
DTI: Diffusion-tensor imaging
IRB: Institutional review board
RPQ: Rivermead post-concussion syndrome questionnaire
PDS: Post-traumatic diagnostic scale
DSM: Diagnostic and statistical manual of mental disorders
IQR: Inter-quartile range
GLMM: Generalized linear mixed models
SD: Standard deviation
MCI: Mild cognitive impairment
ADHD: Attention deficit hyperactivity disorder
EPI: Echo-planar imaging
FA: Fractional anisotropy
MD: Mean diffusivity
RD: Radial diffusivity
AD: Axial diffusivity
IFOF: Inferior fronto-occipital fasciculus
SLF: Superior longitudinal fasciculus
